# Cor Triatriatum Sinister in Adulthood: A Rare Clinical Encounter

**DOI:** 10.7759/cureus.87108

**Published:** 2025-07-01

**Authors:** Aakash Rana, Jack Xu, Mark Mitchell

**Affiliations:** 1 Medicine, Central Arkansas Veterans Healthcare System, Little Rock, USA; 2 Cardiology, Novant Health, Winston-Salem, USA

**Keywords:** atrial septal defect, congenital abnormality, cor triatriatum sinister, patent foramen ovale (pfo), pulmonary venous obstruction

## Abstract

Cor triatriatum sinister (CTS) is a rare congenital anomaly characterized by a fibromuscular membrane that divides the left atrium into proximal and distal chambers. Typically, the pulmonary veins drain into the proximal chamber, while the distal chamber remains continuous with the left atrial appendage and the mitral valve. Communication between the two chambers occurs through one or more fenestrations of variable size. We report the case of a 61-year-old woman with a history of atrial flutter status post-ablation and atrial septal defect repair in infancy, who was incidentally found to have CTS on multimodal imaging. In the absence of hemodynamic compromise or CTS-related symptoms, conservative management was deemed appropriate. This case highlights the value of contemporary imaging modalities in the incidental detection of congenital anomalies in adults and emphasizes the need for individualized management strategies.

## Introduction

Cor triatriatum sinister (CTS) is a rare congenital abnormality, occurring in approximately 0.1-0.4% of patients with congenital heart disease [1]. In CTS, the left atrium (LA) is anatomically divided into proximal and distal chambers by a fibromuscular membrane [2]. The pulmonary veins typically drain into the upper, accessory LA, while the lower, true LA communicates with the left atrial appendage (LAA) and the mitral valve. These two compartments are connected through one or more openings of variable size and number [3].

CTS can present in either a classical or atypical form. The classical form is characterized by a solitary thin membrane within the LA, whereas the atypical form is associated with additional cardiac abnormalities [4]. Patients with CTS often present with pulmonary venous obstruction and pulmonary arterial hypertension, which may progress to congestive heart failure. The severity of clinical manifestations depends on several factors, including the size of the fenestration, the pressure gradient between the proximal and distal LA chambers, and the presence of associated congenital anomalies such as atrial septal defect (ASD), patent foramen ovale, or anomalous pulmonary venous return [5].

When the membrane is incomplete or contains large fenestrations, the condition may remain asymptomatic and is often diagnosed incidentally in adulthood. We present the case of a 61-year-old woman with a history of atrial flutter status post-ablation and ASD repair at 18 months of age, who was incidentally found to have CTS on imaging and was managed conservatively.

## Case presentation

**Figure 1:**
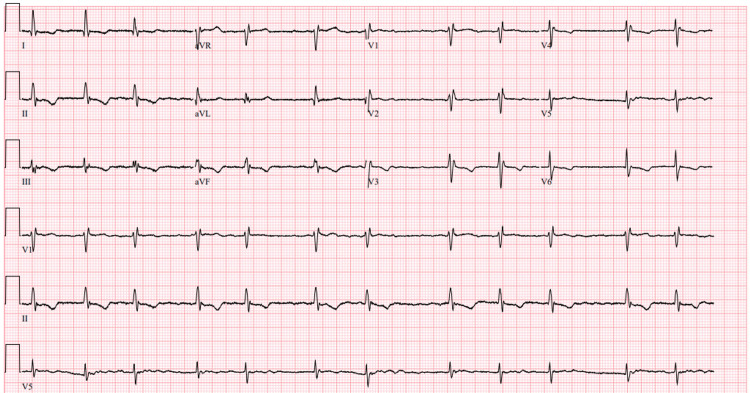
Pre-procedure ECG showing rate-controlled atrial fibrillation with chronic T-wave inversions in leads V2-V6, II, III, and aVF

The patient was a 61-year-old female with a past medical history of persistent atrial fibrillation on apixaban, typical atrial flutter status post-ablation, ASD repair at 18 months of age, asthma, and type 1 diabetes mellitus. She presented for outpatient pulmonary vein isolation ablation for persistent atrial fibrillation. Her blood pressure was mildly elevated at 165/75 mmHg, with other vital signs remaining stable. On physical examination, heart sounds were irregular. No crackles or wheezes were noted on lung auscultation. Pre-procedure ECG showed rate-controlled atrial fibrillation with T-wave inversions in leads V2-V6, II, III, and aVF (Figure 1).

**Figure 2:**
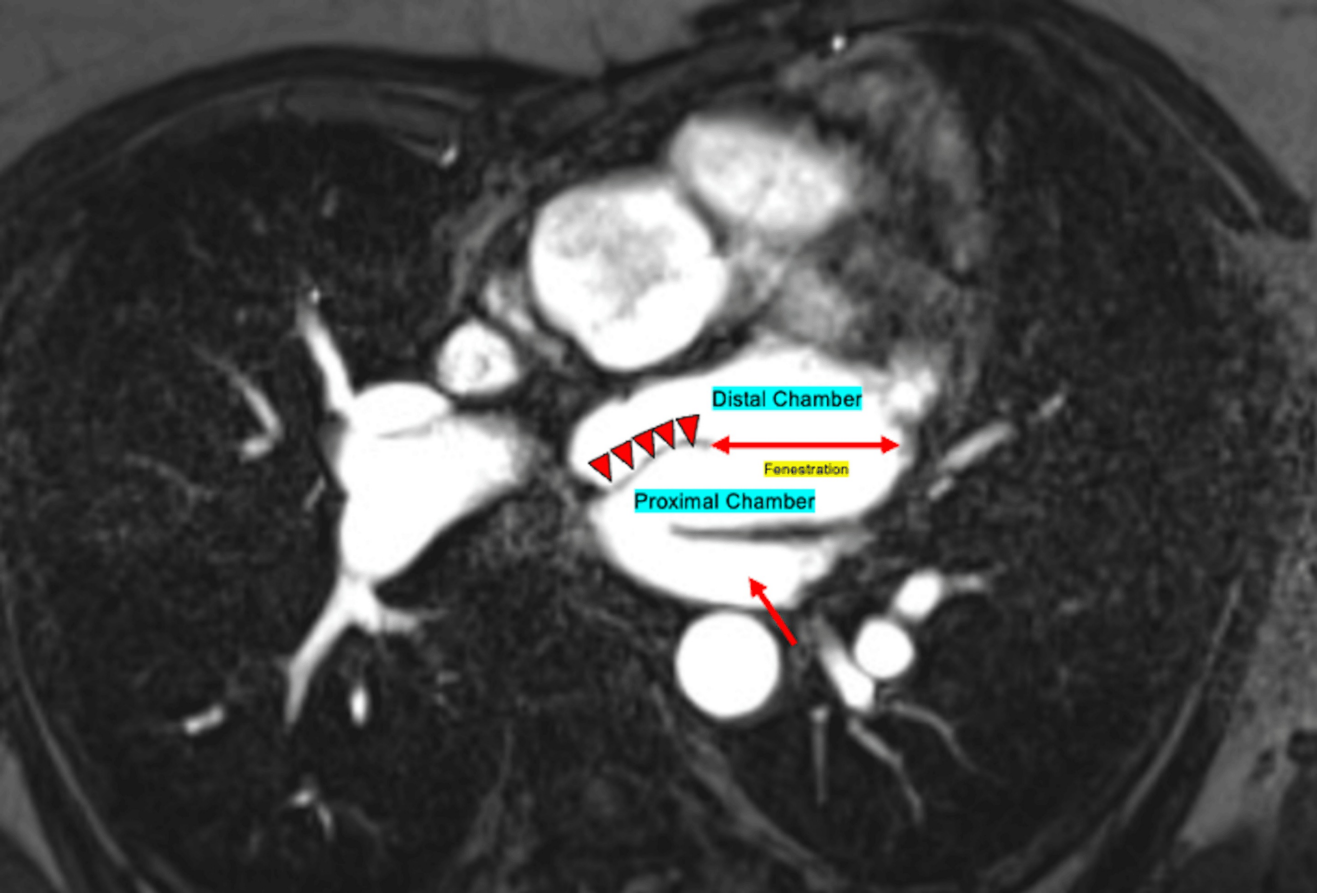
Cardiac MRI showing the LA divided into proximal and distal chambers by an abnormal septum (red arrowheads) The right pulmonary vein drains into the proximal chamber (red single-headed arrow). A fenestration is visible along the left lateral margin (red double-headed arrow). LA, left atrium

A pre-procedure cardiac MRI was obtained a few days prior to the procedure. The cardiac MRI revealed a membrane along the medial margin of the LA, extending from superior to inferior, with a possible fenestration along the left lateral margin. This membrane appeared to partially divide the LA into proximal and distal chambers, with the proximal chamber receiving the pulmonary veins - findings concerning for CTS (Figure 2).

**Figure 3:**
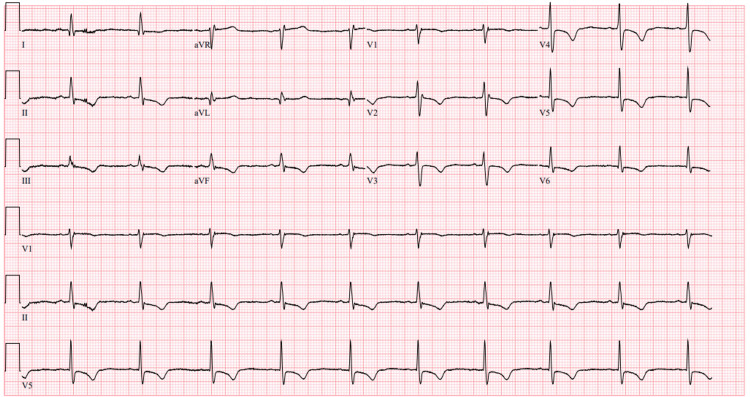
Post-DCCV ECG showing normal sinus rhythm with chronic T-wave inversions in leads V2-V6, II, III, and aVF DCCV, direct current cardioversion

Imaging showed no evidence of thrombus in the LA or LAA. Due to the identification of CTS on cardiac MRI, the planned pulmonary vein isolation ablation was canceled. Instead, the patient was admitted for sotalol loading with QT interval monitoring for management of persistent atrial fibrillation. The following day, she underwent direct current cardioversion (DCCV), successfully restoring normal sinus rhythm. Post-DCCV ECG demonstrated normal sinus rhythm with T-wave inversions in leads V2-V6, II, III, and aVF (Figure 3).

The patient was discharged from the hospital on sotalol 120 mg twice daily, with plans for further outpatient evaluation of CTS, as she remained asymptomatic at the time. She was seen in the outpatient clinic one month after discharge for evaluation of chest tightness that occurred shortly after exertion. Coronary CT angiography (CCTA) was performed as part of the ischemia workup and showed no evidence of coronary plaque or stenosis. The CCTA findings were consistent with the previous cardiac MRI, and the prior ASD repair was also visualized (Video 1).

**Video 1:** CCTA showing the LA divided into proximal and distal chambers by an abnormal septum located along the medial margin of the atrium Both pulmonary veins drain into the proximal chamber. A fenestration is visible in the left lateral margin. Evidence of ASD repair is also noted. ASD, atrial septal defect; CCTA, coronary CT angiography; LA, left atrium

**Figure 4:**
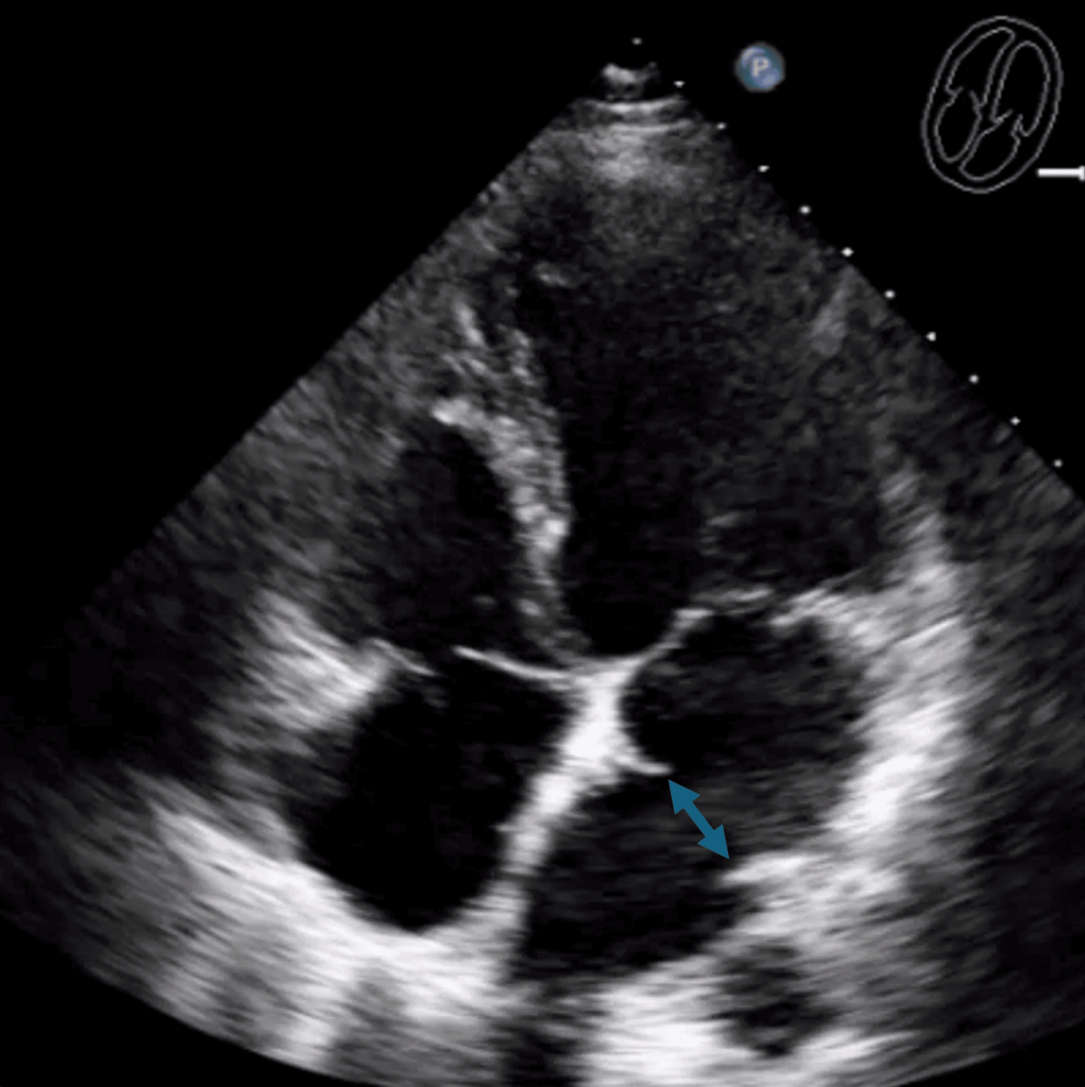
TTE showing an abnormal septum within the LA (double-headed arrow) LA, left atrium; TTE, transthoracic echocardiogram

A transthoracic echocardiogram (TTE) was obtained, showing a left ventricular ejection fraction of 50-55% and an abnormal septum within the LA (Figure 4).

Color Doppler imaging showed no evidence of mitral valve regurgitation or stenosis, and no pressure gradient was observed within the LA (Video 2).

**Video 2:** Color Doppler on TTE showing no evidence of mitral valve regurgitation or stenosis and no pressure gradient within the LA LA, left atrium; TTE, transthoracic echocardiogram

The sotalol dose was reduced from 120 mg twice daily to 60 mg twice daily, resulting in improvement of the patient’s symptoms in the context of underlying asthma, as noted during the follow-up visit. The patient is currently being followed in our clinic with annual surveillance TTE.

## Discussion

As mentioned in the introduction, CTS is a rare congenital anomaly, predominantly reported in pediatric populations, making its presentation in adult patients, such as ours, an uncommon occurrence. CTS has no known association with sex or genetic disorders [6]. The condition is classified according to the number and size of openings within the fibromuscular septum, as described by Loeffler [7]. In Group 1, no fenestrations are present, and the proximal chamber of the LA must drain into the right heart. Group 2 is characterized by a few small fenestrations in the septum, often leading to obstructive symptoms. In Group 3, the proximal and distal chambers communicate freely through a single large fenestration, typically without obstruction [6,7]. Patients in Groups 1 and 2 are often diagnosed during infancy due to significant obstruction, while those in Group 3 are usually asymptomatic and more commonly identified in adulthood.

Several theories have been proposed to explain the etiology of CTS, with the malincorporation theory being the most widely accepted. This theory postulates that CTS results from incomplete incorporation of the common pulmonary vein into the LA during embryological development [6].

CTS is most commonly diagnosed using TTE, although other imaging modalities such as CT and MRI may be employed when echocardiographic findings are inconclusive [6]. While cardiac catheterization was once the standard diagnostic approach, it is now rarely used due to the advent of less invasive and more advanced imaging techniques [8].

Electrocardiographic findings in CTS are typically normal in the absence of obstruction. However, in cases of significant obstruction, ECG may show signs of right ventricular hypertrophy, including right axis deviation and a prominent R or R’ wave in lead V1, often secondary to pulmonary hypertension [9]. Interestingly, some patients with CTS may present with atrial fibrillation, which has been shown to resolve following surgical membrane resection. It remains uncertain whether this improvement is directly related to the removal of the membrane or is a result of alleviating atrial stretch caused by obstructed flow [10].

According to the American Heart Association guidelines for adults with cor triatriatum, all adult patients, regardless of symptomatology, should be evaluated for additional congenital anomalies, particularly atrial or ventricular septal defects. The presence of pulmonary vein abnormalities should also be suspected. In symptomatic patients with a history of prior CTS repair, pulmonary vein stenosis should be investigated. Symptoms attributable to pulmonary venous inflow obstruction or a high-pressure gradient across a restrictive fibromuscular membrane are considered reasonable indications for surgical intervention [11].

In our case, we describe a 61-year-old woman with a history of atrial flutter, status post catheter ablation, and ASD repair at 18 months of age, who was incidentally found to have CTS on MRI (Figure 2). The patient did not report any symptoms of outflow obstruction, such as orthopnea, cyanosis, hemoptysis, or chest pain. She did, however, experience chest tightness after initiation of sotalol, though a complete ischemic workup was negative. The chest tightness was likely related to a high dose of sotalol in the context of asthma, and the symptoms improved with dose reduction [12].

Despite undergoing atrial flutter ablation and septal defect repair in infancy, the patient had no prior diagnosis of CTS. Notably, she remained asymptomatic from CTS, likely due to the presence of a large fenestration that allowed unobstructed flow and the absence of pulmonary vein anomalies. In light of her stable sinus rhythm on sotalol and lack of symptoms, surgical intervention was not pursued at this time. However, if the patient were to experience a recurrence of atrial fibrillation and fail antiarrhythmic therapy, surgical removal of the membrane may be considered, as has been suggested in previous case reports.

## Conclusions

CTS is a rare congenital anomaly, particularly in adults, where it is most often diagnosed incidentally. This case underscores the importance of considering CTS in adults presenting with unexplained atrial arrhythmias or a history of congenital heart repair. The incidental detection of CTS in this 61-year-old woman, alongside the absence of obstructive symptoms and the presence of a large fenestration without associated pulmonary venous anomalies, supports a conservative, nonsurgical management strategy. The case highlights the value of comprehensive cardiac imaging and emphasizes the need for individualized care based on anatomical findings and clinical presentation. Ongoing follow-up is essential, with annual surveillance transthoracic echocardiography recommended, particularly in patients with a history of arrhythmias, as surgical intervention may become necessary if symptoms or rhythm disturbances recur.
